# Artificial intelligence/machine learning and journalology: Challenges and opportunities

**DOI:** 10.1111/aogs.14772

**Published:** 2024-01-29

**Authors:** Prakesh S. Shah, Ganesh Acharya

**Affiliations:** ^1^ Department of Pediatrics Mount Sinai Hospital, University of Toronto Toronto Ontario Canada; ^2^ Department of Clinical Science, Intervention and Technology (CLINTEC) Karolinska Institutet and Center for Fetal Medicine, Karolinska University Hospital Stockholm Sweden; ^3^ Women's Health and Perinatology Research Group UiT – The Arctic University of Norway Tromsø Norway

Disruptions generated by large language models (LLM), commonly known as machine learning (ML) or artificial intelligence (AI), have been knocking on the doors of journals and publishers for some time now. The varying acceptance or rejections by various groups of journals and publishers call for detailed introspection as to what challenges and opportunities lie ahead for the authors, editors, sponsoring organizations, scholarly societies, and publishers of academic journals. The literature is full of words of caution, offering self‐acclaimed wisdom and advising individuals to tread carefully with regard to infiltration of the scientific publishing world by a plethora of AI/ML techniques pioneered by various developers. In this editorial, we explore and identify avenues where nuanced discussions are needed from the entire community to address challenges and opportunities for everyone engaged in scientific publications.

With the availability of LLM, the biggest challenge journals face is how to authenticate validity and quality of research. We do not think this issue emerged solely after the integration of AI/ML tools in the mainstream. The extremely limited ability of expert reviewers, editors, or publishers to authenticate validity and quality of research is a longstanding and known challenge. Until recently, journals relied upon authors themselves, with some oversight from their academic institutions, to confirm integrity and reliability of authors' work. The advent of AI/ML makes it relatively “easy” to produce an output using very little “human intelligence”. At times, it has been shown to produce a completely counterfeit report of a made‐up scientific inquiry, making it very difficult to discern from a real output.^1^ Even though tools to detect AI generated text, such as Turnitin AI detector,^2^ GPTZero,^3^ and others are available, this may threaten the transparency we always aim for in scientific publications. This has worried the scientific community about how to protect academic journals from systematic manipulation of the publication process, and responses from different journals have been variable. Some have put an outright call for complete rejection of any output produced with AI/ML assistance and, on the other hand, others have allowed the Chat Generative Pre‐trained Transformer (ChatGPT)^4^ to be on the author list. With such diverse opinions, the issue has become urgent. Some guidelines have been suggested by the International Committee of Medical Journal Editors and the World Association of Medical Editors. The essence of these guidelines is that the authors can use AI/ML/LLM tools for various purposes in preparation of their articles. This includes editing, polishing, linguistic enhancement, grammar check, spelling, reviewing background material, and contextualizing (except for generating text). However, they should transparently report and acknowledge the use of these techniques. Specific inputs such as video or image manipulations are not permitted as they can again distort the factual aspects of inquiry. As per current understanding, there seems to be a consensus that chatbots should not be authors of a scientific publication because they cannot take public responsibility for their work, approve the final version of the manuscript, “understand” the conflict‐of‐interest statement to sign it, and be held accountable for integrity and accuracy of the scientific work. *Acta Obstetricia et Gynecologica Scandinavica* (AOGS) supports these recommendations; however, further thoughts and perspectives will be needed as things evolve.

In various forms, most journals, including editors and publishers, have started leveraging “computer expertise”—a form of machine learning; for example, to check for plagiarism. Some journals have taken the next step by allowing machines to detect deviations from their standard requirements, such as number of words, subheadings needed, the organization of the article, and so on. A thought is also percolated for the use of LLM for peer review of articles; however, conceptualizing the idea of machines to peer review an article potentially generated by machines sends some quivers. The use of LLMs in editorial workflows could be explored; however, further discussions with data scientists and experts would be needed to recognize what aspects of specialized technical expertise to be employed. Opportunities that can arise from AI/ML techniques to editorial workflow include primary paper review, reviewer selection, automation of language editing, proofreading, compliance checks for manuscript's formatting requirements, and assistance to editors to enhance efficiency and timeliness of manuscript handling.^5^ However, editors, publishers, and even authors find it challenging to clearly discern abstracts or articles generated by LLM from the ones written by human authors. This brings back the concerns that many have with regards to biases introduced by such models, lack of understanding of mechanics behind result generation, and potential misuse of technology. Furthermore, ethical dilemma related to access and equity may arise as availability of LLM may be restricted to those who can afford it. Editors must exercise caution, ensuring that results generated by LLM are checked for reproducibility, validity, and generalizability. One also needs to be aware of the fact that while ethical codes exist, they may not be embedded within all LLM. Supporting editors to become AI‐literate is important in this regard. Streamlining operation of a journal can improve its content and potentially result in articles that readers find easy to read. However, despite their flexibility and logical approach, LLM can be biased or outdated based on the data and models they have been trained on. They can hallucinate (create fake response when they do not know the answer),^6^ exhibit limited common‐sense reasoning, and are not self‐critical. Therefore, it is undesirable that the entire process of article preparation and processing is run by LLM, and human checks on misrepresentations and biased summaries should not become a lost art.

From the authors' perspectives, LLM offer exciting opportunities for efficiency, speed, and improvement in final products. Currently built in processes like spelling checks and thesaurus are present in the existing computer packages and are commonly used without any negative reactions. In the future, LLM can help authors to update a rejected article to meet another journal's formatting requirements, as some of these “nonscientific” and clerical tasks of article writing are not built to feed a scientist's curiosity. The LLM can process large amount of data and can help generate literature reviews quickly. It can also help in developing clinical pathways, guidelines, or decision supports. Additionally, LLM can expedite the knowledge translation process by creating necessary materials form the available articles, and possibly help personalize care for individual patients based on their characteristics. Generating images and videos can also be accomplished; however, warnings have been issued against their use in a scientific publication.^7^ Discussions and dilemmas surrounding the use of LLM in scientific writing will continue; and we will need to develop an adaptive process for governing their use.

So, where does this leave authors, editors, reviewers, and publishers? Ignoring LLMs completely and being merely amazed by them will not take any journal forward. On the other hand, blindly accepting them in totality would also be a grave mistake. Somewhere along this spectrum is where all interested parties need to agree and start. The stance should be reviewed on a regular basis as advances in ML/AI/LLM will occur faster than one can think. Whatever point in this spectrum a journal or group of journals chose, intelligent oversight on its production with vigilant eyes is a necessity. Transparency regarding their use and acknowledgement of the rigor applied after their use are essential for readers to understand the role of AI/ML. Collectively, society, including academic institutions, scholarly societies and organizations, must ensure that their access to AI/ML is not limited to a select few and that it does not widen the gap within the publishing community. It is important to monitor the fairness, effectiveness, safety, and ethical implications of integrating AI in editorial workflow.

In conclusion, planning, conducting, executing, reporting, disseminating, and translating research into practice are at a critical juncture in history. Gone are the days when the gap between knowledge generation and knowledge translation was approximately 20 years. We have witnessed a narrowing of this gap just by the advent of internet and finger‐tip availability of research conducted anywhere in the world. AI/ML has the capacity to expedite this process even faster. However, the caution everyone emphasizes is to ensure that in this “race to the top”, the pathway that individual researchers use is scientific, reliable, valid and above all true. All parties must come to the table with an open and adaptive mind to ensure that there is no loss of human nuance.^8^ As even ChatGPT stated: “It is important to keep in mind that ChatGPT should always be used in combination with the expertise and judgement of human experts and its output should be validated before it is used in clinical practice.”^9^ A collaborative effort between researchers, academic publishers, healthcare professionals, policymakers, and technology developers to harness AI/ML techniques for the benefit of our patients will be a way forward.^10^
